# Interaction between Obesity and the *NFKB1* - 94ins/delATTG Promoter Polymorphism in Relation to Incident Acute Coronary Syndrome: A Follow Up Study in Three Independent Cohorts

**DOI:** 10.1371/journal.pone.0063004

**Published:** 2013-05-09

**Authors:** Jakob Gerhard Stegger, Erik Berg Schmidt, Tina Landsvig Berentzen, Anne Tjønneland, Ulla Vogel, Eric Rimm, Thorkild I. A. Sørensen, Kim Overvad, Majken K. Jensen

**Affiliations:** 1 Department of Cardiology, Center for Cardiovascular Research, Aalborg Hospital, Aarhus University Hospital, Aalborg, Denmark; 2 Department of Anesthesiology, Aalborg University Hospital, Aalborg, Denmark; 3 Institute of Preventive Medicine, Copenhagen University Hospital, Copenhagen, Denmark; 4 Institute of Cancer Epidemiology, Danish Cancer Society, Copenhagen, Denmark; 5 National Research Centre for the Working Environment, Copenhagen, Denmark; 6 Departments of Nutrition and Epidemiology, Harvard School of Public Health, Boston, Massachusetts, United States of America; 7 Department of Medicine, Channing Division of Network Medicine, Brigham and Women’s Hospital and Harvard Medical School, Boston, Massachusetts, United States of America; 8 The Novo Nordisk Foundation Centre for Basic Metabolic Research, Section on Metabolic Genetics, Faculty of Medical and Health Sciences, University of Copenhagen, Copenhagen, Denmark; 9 Department of Public Health, Section for Epidemiology, Aarhus University, Aarhus, Denmark; INRCA, Italy

## Abstract

**Introduction:**

The NF-κB transcription factor family regulates several genes encoding pro-inflammatory and anti-inflammatory proteins in adipose tissues and in atherosclerotic plaques. The deletion variant allele of the *NFKB1* - 94ins/delATTG promoter polymorphism leads to lower transcript levels of the p50 subunit, and the variant allele has been associated with the risk of several inflammatory diseases as well as coronary heart disease where inflammation is important in the pathogenesis. The objective of this study was to explore the potential interaction between the *NFKB1*-94ins/delATTG promoter polymorphism and general, abdominal, and gluteofemoral obesity in relation to the risk of incident acute coronary syndrome (ACS) in three large independent cohorts.

**Methods and Results:**

The analyses were conducted in the Danish prospective study Diet, Cancer and Health and the two US based cohorts; Nurses’ Health Study and Health Professionals Follow-up Study. We conducted sex stratified analyses that included 1202 male and 708 female cases of incident ACS. We observed a positive association for general and abdominal obesity with risk of incident ACS, independent of genotype in both genders. Gluteofemoral obesity was negatively associated with ACS risk in women independent of genotype, whereas there was no clear association for men. We calculated the relative excess risk due to interaction (RERI) and observed a statistically significant excess risk among men jointly exposed to general or abdominal obesity and the variant allele of the *NFKB1*-94ATTG polymorphism, whereas there was a tendency towards sub-additivity for gluteofemoral obesity. The excess risks in all analyses were, however, small and could not clearly be demonstrated in women.

**Conclusion:**

The variant allele of the *NFKB1*-94ins/delATTG promoter polymorphism did not substantially modify the association between obesity and incident ACS.

## Introduction

Obesity is associated with a higher risk of acute coronary syndrome (ACS)[1]–[4]. However, many obese do not experience ACS, and other factors may affect the implications of obesity.

Adipose tissue is, in addition to being a storage organ for energy, an active endocrine organ that secretes both pro- and anti-inflammatory cytokines [5]. As obesity develops, the release of adipose tissue derived cytokines increases [6], and the resulting state of chronic low-grade inflammation may accelerate the atherosclerotic processes in the arterial wall, and thus lead to the increased risk of ACS[6]–[8].

Many genes encoding pro-inflammatory and some genes encoding anti-inflammatory proteins in both adipose tissues and atherosclerotic plaques are regulated by the NF-κB transcription factor family[6], [9]–[11]. The NF-κB family consists of heterodimeric or homodimeric combinations of five subunits (p50, p100, RelA(p65), RelB and c-Rel), and different combinations have different target genes. The p50/p50 homodimer represses transcription of pro-inflammatory cytokines like TNFalpha and IL-12 and promotes transcription of the anti-inflammatory IL-10 [10], [11].

The *NFKB1*–94ins/delATTG promoter polymorphism is an insertion/deletion of four bases in the promoter region of the *NFKB1* gene encoding the p50 subunit, and the variant allele containing the deletion produces lower transcript levels of the p50 subunit [12]. The *NFKB1* - 94ins/delATTG promoter polymorphism is the only functional polymorphism in *NFKB1* [12] and the polymorphism has been associated with both the inflammatory marker CRP and diseases where inflammation is important in the diseases pathogenesis including ACS[12]–[14]. The *NFKB1* - 94ins/delATTG promoter polymorphism is not a single nucleotide polymorphism (SNP) and thus not detected directly by genome wide association scans. However, the *NFKB1* - 94ins/delATTG promoter polymorphism is in high linkage disequilibrium with rs230529 and rs4699030 (r^2^ = 0.741 and 0.714, respectively) [15], but as indicated by the r^2^ of 0.7 the SNPs were not in complete linkage disequilibrium with the *NFKB1* - 94ins/delATTG promoter polymorphism.

The objective of this study was to explore the potential biologic interaction between the *NFKB1*–94ins/delATTG promoter polymorphism and obesity in relation to the risk of incident ACS.

## Results

Baseline characteristics of the study participants are presented in Table 1. Compared to the controls both male and female cases in DCH appeared on average older, had a shorter education, fewer were never smokers, and drank less alcohol. In NHS and HPFS, age and smoking status were matching factors, and thus no differences were observed, but as in DCH, cases drank less alcohol than controls. In all studies, cases had a higher prevalence of hypertension, diabetes mellitus and hypercholesterolemia at baseline. Only few were current smokers in HPFS compared to NHS and DCH, and there were higher prevalence’s of hypertension, hypercholesterolemia and diabetes mellitus in the two US cohorts.

**Table 1:** Baseline characteristics of controls and cases of acute coronary syndrome.

	Diet, Cancer and Health					US cohorts			
	Men			Women		HPFS (men)		NHS (women)	
	Controls	Cases	Controls		Cases	Controls	Cases	Controls	Cases
Variable	(*n* = 922)	(*n* = 775)	(*n* = 797)		(*n* = 237)	(*n* = 873)	(*n* = 427)	(*n* = 922)	(*n* = 471)
Age	56.0 (51;63)	57.9 (52;64)	55.9 (51;63)		59.9 (52;64)	64.3 (52;76)	64.2 (51;75)	59.9 (50;67)	60.1 (51;68)
Educational level									
Basic school	10.7%	15.1%	21.5%		31.7%	0%	0%	0%	0%
Higher education 1–2 years	13.5%	17.2%	31.2%		32.9%	0%	0%	69.7%	68.2%
Higher education 3–4 years	40.9%	40.4%	36.3%		29.1%	0%	0%	18.9%	19.1%
Higher education >4 years	34.9%	27.4%	11.0%		6.3%	100%	100%	9.1%	6.4%
Postmenopausal	n/a	n/a	58.1%		70.9%	n/a	n/a	87.5%	89.0%
Hormonal replacement therapy	n/a	n/a	31.4%		25.3%	n/a	n/a	37.4%	33.8%
Smoking status									
Never smoker	26.5%	14.2%	42.0%		24.5%	41.2%	40.8%	36.2%	35.2%
Former smoker	34.9%	27.0%	21.6%		16.5%	50.3%	49.7%	38.1%	38.4%
Current	38.6%	58.8%	36.4%		59.1%	8.5%	9.6%	25.7%	26.3%
Alcohol (g/day)	19.9 (3.1;61.9)	16.6 (2.0;60.9)	8.8 (1.1;34.4)		6.0 (0.7;32.6)	13.1 (0.0;36.5)	10.3 (0.0;32)	6.1 (0.0;16.4)	4.3 (0.0;14.0)
Hypertension^1^	13.9%	22.5%	16.1%		39.7%	29.0%	37.0%	28.3%	51.0%
Diabetes Mellitus^2^	2.6%	5.0%	1.0%		5.5%	3.6%	9.6%	6.4%	15.1%
Hypercholesterolemia^3^	9.4%	12.3%	5.9%		17.3%	40.6%	48.5%	40.9%	53.5%

Medians with 10th and 90th percentiles in brackets for continuous variables. Percentages for discrete variables. Diet, Cancer and Health, Health Professionals Follow-up Study (HPFS), and Nurses’ Health Study (NHS).

^1^Self reported hypertension or reporting to use blood pressure lowering medication.

^2^Self reported or reporting to use antidiabetic medications.

^3^Self reported hypercholesterolemia or reporting to use cholesterol lowering medication.

The primary anthropometric exposure variables are presented in Table 2. Among the controls the minor allele (the deletion variant) frequency was 37%–39%, and the *NFKB1* genotype distribution was in Hardy-Weinberg equilibrium in all three studies (data not shown).

**Table 2:** Distribution of exposure variables according to case status.

	Diet, Cancer and Health				US cohorts			
	Men		Women		HPFS (men)		NHS (women)	
	Controls	Cases	Controls	Cases	Controls	Cases	Controls	Cases
Variable	(*n* = 922)	(*n* = 775)	(*n* = 797)	(*n* = 237)	(*n* = 873)	(*n* = 427)	(*n* = 922)	(*n* = 471)
								
Body Mass Index (kg/m^2^)	26.4 (23;31)	27.0 (23;32)	24.6 (21;31)	26.2 (21;33)	25.6 (22;30)	26.1 (22;30)	25.1 (20;31)	26.6 (21;35)
BMI grouped according to WHO								
Underweight	0.3%	0.1%	1.4%	1.3%	1.8%	1.6%	6.8%	6.2%
Normalweight	33%	27%	53%	38%	44%	36%	51%	40%
Overweight	51%	52%	33%	38%	44%	52%	29%	29%
Obese	16%	22%	13%	22%	11%	11%	13%	25%
Waist Circumference (cm)	95 (85;108)	97 (87;112)	80 (69;97)	86 (71;101)	98 (86;110)	99 (87;112)	79 (76;91)	82 (69;99)
WC grouped according to WHO								
Female <88 cm	n/a	n/a	74%	58%	n/a	n/a	84%	72%
Female ≥88 cm	n/a	n/a	26%	42%	n/a	n/a	16%	28%
Male <102 cm	74%	65%	n/a	n/a	72%	66%	n/a	n/a
Male ≥102 cm	26%	35%	n/a	n/a	28%	34%	n/a	n/a
Hip Circumference (cm)	100 (93;108)	101 (93;110)	101 (92;113)	101 (91;114)	103 (94;112)	104 (94;114)	100 (89;112)	103 (91;119)
*NFKB1*-94ATTG polymorphism								
Wildtype/Wildtype	40% (371)	35% (268)	38% (301)	38% (89)	39% (344)	36% (154)	39% (362)	39% (183)
Wildtype/Variant	45% (418)	48% (372)	47% (374)	47% (112)	45% (389)	51% (216)	45% (412)	45% (213)
Variant/Variant	14% (133)	17% (135)	15% (122)	15% (36)	16% (140)	13% (57)	16% (148)	16% (75)

Medians with 10th and 90th percentiles in brackets for continuous variables. Absolute numbers and/or percentages for discrete variables. Diet, Cancer and Health, Health Professionals Follow-up Study (HPFS), and Nurses’ Health Study (NHS).

Note: Sample size in NHS and HPFS with data available on waist and hip circumfererence was lower (426/679 and 382/821, resspectively).

Both general obesity, measured as BMI, and abdominal obesity, measured as WC, were positively associated with ACS independent of genotype. Carrier status did not have a consistent effect on ACS risk among the lean reference groups, but the risk of ACS was highest among the jointly exposed to both the variant allele and obesity (Table 3 and Table 4). Due to the negative association between gluteofemoral obesity measured as HC and ACS, the HC variable was inverted, and the group with the highest HC was the reference group (Table 5). In the lean men, the variant allele was associated with a higher risk of ACS in DCH and in the pooled estimates, but there was no association among the lean females. Gluteofemoral obesity was negatively associated with ACS risk among women with no consistent effect of genotype, whereas we observed no clear association in men.

**Table 3:** RR* with 95% confidence interval in brackets for the combined effect of general obesity and the NFKB1-94ATTG polymophism in relation to acute coronary syndrome.

MEN		Diet, Cancer and Health^1^		HPFS^2^		Metaanalysis				
		*NFKB1*		*NFKB1*		*NFKB1*				
Variable	wt/wt	wt/var	var/var	wt/wt	wt/var	var/var	wt/wt	wt/var	var/var	
Body mass index										
<25 kg/m^2^	1.00 (ref.)	1.14 (0.85; 1.53)	1.10 (0.73; 1.66)	1.00 (ref.)	1.04 (0.72; 1.50)	0.79 (0.46; 1.35)	1.00 (ref.)	1.10	(0.88; 1.38) 0.97 (0.70; 1.35)	
25–29 kg/m^2^	1.15 (0.86; 1.52)	1.40 (1.07; 1.84)	1.71 (1.24; 2.37)	1.21 (0.80; 1.85)	1.65 (1.11; 2.44)	1.07 (0.60;1.89)	1.17(0.92;1.48)	1.48	(1.18; 1.84) 1.52 (1.15; 2.02)	
>29 kg/m^2^	1.49 (1.08;2.05)	1.61 (1.20;2.18)	1.98 (1.35;2.91)	1.04 (0.59;1.82)	1.83 (1.11;3.04)	1.50 (0.68;3.27)	1.36 (1.03;1.80)	1.67	(1.29; 2.15) 1.88 (1.33; 2.65)	
**WOMEN**		**Diet, Cancer and Health^1^**		**NHS^2^**		**Metaanalysis**				
		***NFKB1***		***NFKB1***		***NFKB1***				
**Variable**	**wt/wt**	**wt/var**	**var/var**	**wt/wt**	**wt/var**	**var/var**	**wt/wt**	**wt/var**		**var/var**
Body mass index										
<24 kg/m^2^	1.00(ref.)	0.96(0.59;1.55)	0.69(0.32;1.47)	1.00(ref.)	0.97(0.66;1.42)	1.02(0.60;1.76)	1.00(ref.)	0.96		(0.71; 1.30) 0.90 (0.58; 1.39)
24–28 kg/m^2^	1.15(0.67;1.97)	1.25(0.80;1.97)	0.91(0.44;1.86)	1.23(0.79;1.91)	1.22(0.81;1.84)	1.31(0.77;2.23)	1.20(0.85;1.69)	1.24		(0.91; 1.67) 1.15 (0.75; 1.77)
>28 kg/m^2^	1.47 (0.88;2.45)	1.79 (1.10;2.89)	2.43 (1.35;4.37)	1.67 (1.08;2.58)	2.08 (1.34;3.22)	1.59 (0.84;3.03)	1.58 (1.14;2.21)	1.94		(1.41; 2.69) 2.00 (1.30; 3.09)

Diet, Cancer and Health, Health Professionals Follow-up Study (HPFS), and Nurses Health Study (NHS).

^1^Adjusted for age, smoking status, alcohol consumption, physical activity, and educational level. Women also adjusted for menopausal status.

^2^Adjusted for age, smoking status, alcohol consumption, and physical activity. Women also adjusted for menopausal status and hormone replacement therapy.

* RR estimated by Cox proportional hazards regression in DCH and logistic regression in NHS and HPFS.

**Table 4:** RR* with 95% confidence interval in brackets for the combined effect of abdominal obesity and the NFKB1-94ATTG polymophism in relation to acute coronary syndrome.

MEN	Diet, Cancer and Health^1^			HPFS^2^			Metaanalysis		
	*NFKB1*			*NFKB1*			*NFKB1*		
Variable	wt/wt	wt/var	var/var	wt/wt	wt/var	var/var	wt/wt	wt/var	var/var
Waist circumference									
<94 cm	1.00(ref.)	1.09(0.84;1.42)	1.04(0.71;1.52)	1.00(ref.)	1.05(0.68;1.63)	0.84(0.45;1.59)	1.00(ref.)	1.08	(0.86; 1.35) 0.98 (0.71; 1.36)
94–102 cm	1.04(0.78;1.38)	1.29(0.99;1.69)	1.56(1.14;2.15)	1.35(0.83;2.18)	1.17(0.74;1.85)	0.77(0.37;1.59)	1.11(0.87;1.42)	1.26	(1.00; 1.59) 1.39 (1.04; 1.86)
>102 cm	1.37 (0.98;1.91)	1.55 (1.12;2.15)	2.00 (1.35;2.95)	0.98 (0.56;1.71)	1.54 (0.94;2.54)	1.10 (0.58;2.08)	1.25 (0.94;1.67)	1.55	(1.18; 2.03) 1.70 (1.22; 2.37)
**WOMEN**	**Diet, Cancer and Health^1^**			**NHS^2^**			**Metaanalysis**		
	***NFKB1***			***NFKB1***			***NFKB1***		
**Variable**	**wt/wt**	**wt/var**	**var/var**	**wt/wt**	**wt/var**	**var/var**	**wt/wt**	**wt/var**	**var/var**
Waist circumference									
<80 cm	1.00(ref.)	0.92(0.57;1.48)	0.84(0.42;1.69)	1.00(ref.)	0.85(0.57;1.27)	0.97(0.57;1.67)	1.00(ref.)	0.88	(0.65; 1.20) 0.92 (0.60; 1.41)
80–91 cm	1.69(0.99;2.89)	1.68(1.03;2.76)	0.96(0.47;1.95)	0.87(0.51;1.48)	0.70(0.41;1.21)	0.72(0.35;1.46)	1.21(0.83;1.77)	1.13	(0.79; 1.63) 0.83 (0.50; 1.37)
>91 cm	2.07 (1.09;3.95)	3.53 (1.96;6.35)	5.50 (2.53;12.0)	1.20 (0.64;2.26)	2.04 (1.05;3.99)	1.45 (0.59;3.57)	1.57 (1.00;2.46)	2.78	(1.79; 4.33) 3.11 (1.73; 5.60)

Diet, Cancer and Health, Health Professionals Follow-up Study (HPFS), and Nurses Health Study (NHS).

^1^Adjusted for age, smoking status, alcohol consumption, physical activity, educational level, and hip circumference. Women also adjusted for menopausal status.

^2^Adjusted for age, smoking status, alcohol consumption, physical activity, and hip circumference. Women also adjusted for menopausal status and hormone replacement therapy.

* RR estimated by Cox proportional hazards regression in DCH and logistic regression in NHS and HPFS.

**Table 5:** RR* with 95% confidence interval in brackets for the combined effect of gluteofemoral obesity and the NFKB1-94ATTG polymophism in relation to acute coronary syndrome.

MEN	Diet, Cancer and Health^1^			HPFS^2^			Metaanalysis				
	*NFKB1*			*NFKB1*			*NFKB1*				
Variable	wt/wt	wt/var	var/var	wt/wt	wt/var	var/var	wt/wt	wt/var			var/var
Hip circumference											
>103.5 cm	1.00(ref.)	1.23(0.93;1.63)	1.56(1.11;2.20)	1.00(ref.)	1.32(0.86;2.01)	1.14(0.65;2.00)	1.00(ref.)	1.26			(0.99; 1.59) 1.43 (1.07; 1.92)
98–103.5 cm	0.96(0.70;1.30)	1.04(0.78;1.38)	1.42(1.02;1.99)	1.39(0.84;2.31)	1.53(0.94;2.50)	0.76(0.37;1.55)	1.06(0.81;1.38)	1.15			(0.90; 1.47) 1.27 (0.94; 1.72)
<98 cm	1.07 (0.77;1.48)	1.25 (0.91;1.71)	0.99 (0.64;1.54)	1.29 (0.75;2.23)	1.16 (0.69;1.96)	0.97 (0.45;2.10)	1.12 (0.85;1.49)	1.23			(0.94; 1.61) 0.98 (0.67; 1.44)
**WOMEN**	**Diet, Cancer and Health^1^**			**NHS^2^**			**Metaanalysis**				
	***NFKB1***			***NFKB1***			***NFKB1***				
**Variable**	**wt/wt**	**wt/var**	**var/var**	**wt/wt**	**wt/var**	**var/var**	**wt/wt**			**wt/var**	**var/var**
Hip circumference											
>104 cm	1.00(ref.)	1.21(0.74;2.00)	1.32(0.63;2.78)	1.00(ref.)	1.30(0.80;2.09)	0.85(0.44;1.63)	1.00(ref.)			1.25	(0.89; 1.77) 1.03 (0.63; 1.68)
98–104 cm	1.29(0.72;2.32)	1.49(0.87;2.54)	1.64(0.88;3.07)	1.01(0.55;1.88)	1.04(0.58;1.89)	1.16(0.53;2.56)	1.15(0.75;1.76)			1.27	(0.85; 1.89) 1.44 (0.88; 2.35)
<98 cm	2.33 (1.27;4.26)	2.05 (1.16;3.62)	1.21 (0.51;2.89)	1.59 (0.91;2.79)	1.15 (0.66;2.00)	1.61 (0.81;3.19)	1.90 (1.26;2.86)			1.52	(1.02; 2.27) 1.44 (0.84; 2.47)

Diet, Cancer and Health, Health Professionals Follow-up Study (HPFS), and Nurses Health Study (NHS).

^1^Adjusted for age, smoking status, alcohol consumption, physical activity, educational level, and waist circumference. Women also adjusted for menopausal status.

^2^Adjusted for age, smoking status, alcohol consumption, physical activity, and waist circumference. Women also adjusted for menopausal status and hormone replacement therapy.

* RR estimated by Cox proportional hazards regression in DCH and logistic regression in NHS and HPFS.

We explored the possible interaction between obesity and carrier status by calculating the relative excess risk due to interaction (RERI) (Table S1). In men the combined effects of the *NFKB1* - 94ins/delATTG promoter polymorphism and general or abdominal obesity showed a tendency towards positive interaction. Likewise, we found a tendency towards negative interaction, i.e. subadditivity, for the combined effect of the *NFKB1* - 94ins/delATTG promoter polymorphism and gluteofemoral obesity. However, the estimates for the excess risk were small and could not clearly be demonstrated in women.

## Discussion

In this large prospective study conducted in three independent cohorts, we observed no substantial interaction between obesity and the *NFKB1* - 94ins/delATTG promoter polymorphism in relation to the risk of incident ACS; however, joint exposure to general or abdominal obesity and the variant allele was associated with the highest risk of ACS.

### Strengths and Limitations

We achieved a high level of endpoint ascertainment as cases were validated by direct review of medical records. Likewise, data on anthropometric exposure variables were collected by trained study technicians in DCH, but in NHS and HPFS anthropometric measures were self reported, which could lead to measurement errors. However, all participants in NHS and HPFS were health workers, and the self reported measures have been validated, albeit there was a tendency towards underestimation of obesity measures [16]. Due to the subtle nature of atherosclerosis, reverse causation in relation to the anthropometric variables was a possibility, but we have previously in the total DCH cohort found similar associations for all anthropometric variables, when we conducted separate analyses in participants who experienced ACS within the first 2 years of follow up and in participants who experienced an ACS event after more than 2 years of follow up [3].

We adjusted for several potential confounders related to the associations between obesity and ACS, but did not include hypertension, diabetes mellitus and hypercholesterolemia in the analyses of the presented results as they can be seen as intermediate variables. Thus, their inclusion in multivariate analyses would restrict the outcome to associations through other pathways; however, analyses without these adjustments could lead to confounding from other causes to these intermediate variables. Crude analyses (Table S2), adjusted analyses (Table 3, 4 and 5) and analyses with additional adjustment for hypertension, diabetes mellitus and hypercholesterolemia (data not shown) provided similar results, and thus we do not believe that residual confounding explains our results.

### General Discussion

The variant allele containing the deletion *NFKB1*-94ins/delATTG promoter polymorphism leads to lower levels of the p50 subunit, and this affects both the availability of the anti-inflammatory p50/p50 NF-κB homodimer and the pro-inflammatory p50/p65 NF-κB heterodimer. However, the combined effect of relatively low levels of both p50/p50 and p50/p65 will be pro-inflammatory, as low p50 levels intuitively will affect the concentration of p50/p50 more than the concentration of p50/p65. Furthermore, the p50/p50 homodimer blocks binding sites for the p65 subunit, and, thus, the level of the p50/p65 heterodimer will be relatively closer to normal, due to the concurrent low levels of p50/p50 [11], [12]. CRP transcription is controlled by the p50 homodimer. We have previously found lower levels of CRP protein in serum from carriers of the del-allele compared to ins-allele carriers, supporting the interpretation that the del-allele causes depletion of p50 homodimer [14]. This is also in line with the findings of increased levels of TNFalpha and IL-12 and reduced levels of IL-10 in *NFKB1* knock-out mice [11]. However, the effect of the polymorphism may vary between different tissues. Abdominal adipose tissue secretes a relatively greater amount of pro-inflammatory cytokines than gluteofemoral adipose tissue that secretes more anti-inflammatory cytokines [5]. Thus, in participants with high HC the net effect of the *NFKB1*-94ins/delATTG promoter polymorphism may be a preferential depletion of anti-inflammatory cytokines, and a reduction in the protective effect of gluteofemoral fat deposits. Likewise, the combined effect of abdominal obesity and homozygote variant carrier status may be supra-additive due an excess secretion of pro-inflammatory cytokines as well as an increased inflammatory response to cytokines in atherosclerotic plaques. Even though our results does show a tendency of positive interaction for abdominal obesity and negative interaction for gluteofemoral obesity, and thus supports the above mentioned biological hypothesis, potential interaction between genetic variation in *NFKB1* and obesity was small and does not seem to be of clinical importance. However, due to the importance of inflammation the development of atherosclerosis further studies could be warranted to clarify the biological implications of genetic variations in genes involved in inflammation in relation to coronary heart disease.

In conclusion, we found no substantial interaction between the *NFKB1*-94ins/delATTG promoter polymorphism and general, abdominal, or gluteofemoral obesity.

## Materials and Methods

### Study Populations

The present study was based on three independent cohorts; the Danish prospective study Diet, Cancer and Health (DCH), and two US based cohorts; the Nurses’ Health Study (NHS) and the Health Professionals Follow-up Study (HPFS). The Diet, Cancer and Health study was approved by the National Committee on Health Research Ethics (journal nr. (KF) 01-345/93) and the Danish Data Protection Agency. Written informed consent was obtained from all participants to search information from medical registers.

All studies have previously been described in detail[17]–[19], and thus only a brief description is presented here.

#### Diet, cancer and health

From November 1993 to May 1997, all men and women aged 50–64 years, born in Denmark, living in the greater Copenhagen or Aarhus areas, and with no previous cancer diagnosis registered in the Danish Cancer Registry were invited to participate in Diet, Cancer and Health (DCH). The study was approved by the National Committee on Health Research Ethics (journal nr. (KF) 01-345/93) and the Danish Data Protection Agency. Written informed consent was obtained from all participants to search information from medical registers.

To preserve biological material the present study was conducted as a case-cohort design based on a sex-stratified subcohort drawn randomly from the entire cohort. After case validation, we excluded participants with an ACS diagnosis prior to baseline, and participants for whom information on one or more variables were missing.

All anthropometric data were collected by trained technicians. Height was measured to the nearest 0.5 cm with the participants standing without shoes. Weight was measured using a digital scale with the participants wearing light clothing and recorded to the nearest 0.1 kg. Waist circumference (WC) was recorded to the nearest 0.5 cm and measured at the narrowest part between the lower rib and the iliac crest.

Blood samples were collected from each participant at baseline, and lymphocytes were isolated and frozen within 2 h. For genotyping, DNA was isolated from frozen lymphocytes from cases and participants in the subcohort and the *NFKB1*-94 ins/del promoter polymorphism was determined as previously described[14], [20]–[22]. Controls were included in each run, and repeated genotyping of a random 10% subset yielded 100% identical genotypes [14].

Information on confounders was obtained at baseline through questionnaires including socio-demographic factors, lifestyle, medication and prevalent disease.

In Diet, Cancer and Health, ACS was defined as unstable angina pectoris and myocardial infarction (ICD-8∶410–410.99 and ICD-10: I20.0, I21.0–I21.9). Potential cases were identified by linkage to the Danish National Patient Registry [23] and the Danish Causes of Death Registry using the Danish Civil Registration System, in which every Danish citizen is identified by a unique 10 digit number. All incident cases between baseline and ultimo 2003 were validated by review of medical records in accordance with the guidelines of the American Heart Association and the European Society of Cardiology for use in epidemiology [24]. Furthermore, participants with a sudden cardiac death diagnosis (ICD 8∶427.27 or ICD 10: I46.0–I46.9) were accepted as cases, if the cardiac arrest at validation was believed to have been caused by ACS.

A total of 80,996 men and 79,729 women were invited, and 27,148 men (34%) and 29,863 women (37%) consented to participate. The sex stratified cohort sample consisted of 1869 participants. During a median follow up time of 6.8 years, 802 male and 250 female cases of incident ACS were diagnosed. After exclusions of participants for whom information was missing on the exposure variables or covariate data, there were 769 male and 224 female cases, and the subcohort comprised 1695 participants. Participants in the subcohort may throughout the paper be referred to as controls, even though the subcohort included 33 participants who had become cases.

#### The nurses’ health study and the health professionals follow-up study

The Nurses’ Health Study (NHS) was established in 1976 at the Channing Laboratory of the Brigham and Women’s Hospital, Boston, US. The study included married, female, registered nurses aged 30–55 years residing in one of 11 US states.

The Health Professionals Follow-up Study (HPFS) was established at the Harvard School of Public Health in 1986 with methods similar to that described for the NHS. It is a longitudinal study among male health workers in the United States aged 40–75 years at enrollment.

In both studies, the participants have received follow-up questionnaires biennially to update information on lifestyle factors, body weight, and newly diagnosed illnesses. Self-reported information on height and weight was obtained at baseline, and self-reported measures of WC and hip circumference (HC) were obtained in 1986 in NHS and 1987 in HPFS, respectively.

In the NHS, blood samples were collected in 1989–1990 from participants free of cardiovascular disease and cancer. Whole blood samples were centrifuged and stored in cryotubes as plasma, buffy coat, and red blood cells. DNA was extracted from the buffy coat fraction of centrifuged blood with the QLAmp Blood Kit (Qiagen, Chatsworth, CA, USA). The genotypes were determined by Taqman SNP allelic discrimination by means of an ABI 7900HT (Applied Biosystems, Foster City, CA, USA)as previously described [14], [25]. Similarly, blood samples were collected from participants from participants free of cardiovascular disease and cancer in the HPFS in 1993–1995.

In both studies, anthropometric (except waist and hip measures which were only obtained once in 1986/1987) and covariate data were derived from questionnaires administered at blood draw (1990 in the NHS and 1994 in the HPFS), with missing information substituted from previous questionnaires [16], [18].

Cases of ACS were in both NHS and HPFS defined as myocardial infarction and fatal coronary heart disease. Potential cases were participants who reported incident events on the follow-up questionnaires and deceased participants. Deaths were identified from state vital records and the National Death Index or reported by the participant’s next of kin or the postal system. Cases were validated primarily through review of medical records by physicians blinded to the participant’s questionnaire reports [26]. The diagnosis of myocardial infarction was confirmed on the basis of the criteria of the World Health Organization, while fatal coronary heart disease was confirmed by an examination of hospital or autopsy records, by the listing of coronary heart disease as the cause of death on the death certificate or if coronary heart disease was the underlying and most plausible cause of death.

For the present study, we used nested case-control designs. Blood was donated from 32,826 Nurses and 18,224 HPFS participants. Between blood draw and June 2004, 512 female incident cases of ACS were identified in NHS, and 454 male cases were identified in HPFS. Using risk-set sampling, controls were selected randomly and matched in a 2∶1 ratio on age, smoking, and month of blood return, among participants who were free of cardiovascular disease at the time ACS was diagnosed in the case patient [27].

After exclusions of participants for whom information was missing on the *NFKB1* genotype, body mass index and covariate data, the NHS comprised 471 cases of incident ACS and 922 controls, and the HPFS comprised 427 cases and 875 controls. However, information on WC and HC was not available for all participants, and thus analyses regarding WC and HC only included 426 cases and 679 controls in NHS and 382 cases and 821 controls in HPFS.

### Statistical Analyses

In accordance with the case-cohort design and to allow for delayed entry of the participants, the incidence rate ratios in DCH were analyzed by weighted Cox’s proportional hazards regression models with age as the underlying time variable [28], [29]. The observation time was censored by death of other causes than ACS, emigration or study end at December 31^st^ 2003. Due to the nested case-control design, incidence rate ratios were estimated by logistic regression models in NHS and HPFS. Crude conditional and unconditional analyses (adjusted for matching factors) showed very similar results (data not shown). However, in the conditional analyses a significant number of cases where missing a match due to missing exposure/covariate information, and thus we chose to provide results from unconditional analyses.

We used body mass index (BMI, calculated as the weight in kilograms divided by the square of the height in meters) as a measure of general obesity, and body fat distribution was expressed as WC and HC mutually adjusted describing abdominal and gluteofemoral obesity, respectively. The distribution of anthropometric measures were described in tables according to the WHO classification, but to achieve the best possible statistical power, measures of obesity were included in the analyses as categorical variables with 3 levels according to the distribution among cases with the DCH cohort as reference (Men: BMI <25, 25–29 and >29 kg/m^2^; WC <94, 94–102 and >102 cm; HC <98, 98–103.5 and >103.5 cm. Women: BMI <24, 24–28 and >28 kg/m^2^; WC <80, 80–91 and >91 cm; HC <98, 98–104 and >104 cm).

In DCH, we performed separate analyses in men and women and adjusted for potential confounding from age, smoking status, alcohol intake, physical activity (expressed as hours per week of strenuous activities) educational level, and for women menopausal status. In NHS and HPFS we adjusted for age, smoking status, alcohol intake, and physical activity (expressed in terms of metabolic equivalent (MET)-hours). In NHS we furthermore adjusted for menopausal status and hormone replacement therapy. Hypertension, diabetes mellitus, and hypercholesterolemia are possible intermediate variables in analyses of anthropometry and ACS, and therefore, they were not included in the main analyses.

Deviation from an additive model was explored as the relative excess risk due to interaction (RERI) and calculated as suggested by Rothman [30] and Hosmer and Lemeshow [31].

To combine the estimates from the three study populations, we calculated the weighted average of the log rate ratios with weights according to the study-specific variance. A similar model on original scale was used to calculate the combined estimates of the relative excess risk due to interaction.

The proportionality assumptions of the Cox proportional hazards models were evaluated graphically by log-minus-log plots. All analyses were performed using Stata version 11.2 (StataCorp LP, College Station, TX) and SAS version 9 (SAS Institute Inc., Cary, NC).

## Supporting Information

Table S1 RR* with 95% confidence interval in brackets for the combined effect of obesity and the NFKB1-94ATTG polymophism in relation to acute coronary syndrome. Diet, Cancer and Health, Health Professionals Follow-up Study (HPFS), and Nurses Health Study (NHS).
(XLS)

Table S2 Crude analyses. RR * with 95% confidence interval in brackets for the combined effect of obesity and the NFKB1-94ATTG polymophism in relation to acute coronary syndrome. Diet, Cancer and Health, Health Professionals Follow-up Study (HPFS), and Nurses Health Study (NHS).
(XLS)

## References

[1] Bogers RP, Bemelmans WJ, Hoogenveen RT, Boshuizen HC, Woodward M, et al (2007) Association of overweight with increased risk of coronary heart disease partly independent of blood pressure and cholesterol levels: A meta-analysis of 21 cohort studies including more than 300 000 persons. Arch Intern Med 167: 1720–1728. 17846390 10.1001/archinte.167.16.1720

[2] Canoy D (2008) Distribution of body fat and risk of coronary heart disease in men and women. Curr Opin Cardiol 23: 591–598. 18830075 10.1097/HCO.0b013e328313133a

[3] Stegger JG, Schmidt EB, Obel T, Berentzen TL, Tjonneland A, et al (2011) Body composition and body fat distribution in relation to later risk of acute myocardial infarction: A danish follow-up study. Int J Obes (Lond) 35: 1433–1441. 21285940 10.1038/ijo.2010.278

[4] Yusuf S, Hawken S, Ounpuu S, Dans T, Avezum A, et al (2004) Effect of potentially modifiable risk factors associated with myocardial infarction in 52 countries (the INTERHEART study): Case-control study. Lancet 364: 937–952. 15364185 10.1016/S0140-6736(04)17018-9

[5] Kershaw EE, Flier JS (2004) Adipose tissue as an endocrine organ. J Clin Endocrinol Metab 89: 2548–2556. 15181022 10.1210/jc.2004-0395

[6] Hajer GR, van Haeften TW, Visseren FL (2008) Adipose tissue dysfunction in obesity, diabetes, and vascular diseases. Eur Heart J 29: 2959–2971. 18775919 10.1093/eurheartj/ehn387

[7] Ridker PM, Morrow DA (2003) C-reactive protein, inflammation, and coronary risk. Cardiol Clin 21: 315–325. 14621448 10.1016/s0733-8651(03)00079-1

[8] Ross R (1999) Atherosclerosis–an inflammatory disease. N Engl J Med 340: 115–126. 9887164 10.1056/NEJM199901143400207

[9] de Winther MP, Kanters E, Kraal G, Hofker MH (2005) Nuclear factor kappaB signaling in atherogenesis. Arterioscler Thromb Vasc Biol 25: 904–914. 15731497 10.1161/01.ATV.0000160340.72641.87

[10] Pereira SG, Oakley F (2008) Nuclear factor-kappaB1: Regulation and function. Int J Biochem Cell Biol 40: 1425–1430. 17693123 10.1016/j.biocel.2007.05.004

[11] Cao S, Zhang X, Edwards JP, Mosser DM (2006) NF-kappaB1 (p50) homodimers differentially regulate pro- and anti-inflammatory cytokines in macrophages. J Biol Chem 281: 26041–26050. 16835236 10.1074/jbc.M602222200 PMC2642587

[12] Karban AS, Okazaki T, Panhuysen CI, Gallegos T, Potter JJ, et al (2004) Functional annotation of a novel NFKB1 promoter polymorphism that increases risk for ulcerative colitis. Hum Mol Genet 13: 35–45. 14613970 10.1093/hmg/ddh008

[13] Van der Heiden K, Cuhlmann S, Luong le A, Zakkar M, Evans PC (2010) Role of nuclear factor kappaB in cardiovascular health and disease. Clin Sci (Lond) 118: 593–605. 20175746 10.1042/CS20090557

[14] Vogel U, Jensen MK, Due KM, Rimm EB, Wallin H, et al (2011) The NFKB1 ATTG ins/del polymorphism and risk of coronary heart disease in three independent populations. Atherosclerosis 219: 200–204. 21726863 10.1016/j.atherosclerosis.2011.06.018 PMC3827022

[15] Liou YJ, Wang HH, Lee MT, Wang SC, Chiang HL, et al (2012) Genome-wide association study of treatment refractory schizophrenia in han chinese. PLoS One 7: e33598. 22479419 10.1371/journal.pone.0033598 PMC3313922

[16] Rimm EB, Stampfer MJ, Colditz GA, Chute CG, Litin LB, et al (1990) Validity of self-reported waist and hip circumferences in men and women. Epidemiology 1: 466–473. 2090285 10.1097/00001648-199011000-00009

[17] Tjonneland A, Olsen A, Boll K, Stripp C, Christensen J, et al (2007) Study design, exposure variables, and socioeconomic determinants of participation in diet, cancer and health: A population-based prospective cohort study of 57,053 men and women in denmark. Scand J Public Health 35: 432–441. 17786808 10.1080/14034940601047986

[18] Rimm EB, Giovannucci EL, Stampfer MJ, Colditz GA, Litin LB, et al.. (1992) Reproducibility and validity of an expanded self-administered semiquantitative food frequency questionnaire among male health professionals. Am J Epidemiol 135: 1114–26; discussion 1127–36. 10.1093/oxfordjournals.aje.a116211 1632423

[19] Colditz GA, Manson JE, Hankinson SE (1997) The nurses’ health study: 20-year contribution to the understanding of health among women. J Womens Health 6: 49–62. 9065374 10.1089/jwh.1997.6.49

[20] Vogel U, Segel S, Dethlefsen C, Tjonneland A, Saber AT, et al (2010) Associations between COX-2 polymorphisms, blood cholesterol and risk of acute coronary syndrome. Atherosclerosis 209: 155–162. 19748095 10.1016/j.atherosclerosis.2009.08.036

[21] Vogel U, Segel S, Dethlefsen C, Tjonneland A, Saber AT, et al (2009) PPARgamma Pro12Ala polymorphism and risk of acute coronary syndrome in a prospective study of danes. BMC Med Genet 10: 52. 19500413 10.1186/1471-2350-10-52 PMC2698834

[22] Miller SA, Dykes DD, Polesky HF (1988) A simple salting out procedure for extracting DNA from human nucleated cells. Nucleic Acids Res 16: 1215. 3344216 10.1093/nar/16.3.1215 PMC334765

[23] Andersen TF, Madsen M, Jorgensen J, Mellemkjoer L, Olsen JH (1999) The danish national hospital register. A valuable source of data for modern health sciences. Dan Med Bull 46: 263–268. 10421985

[24] Joensen AM, Jensen MK, Overvad K, Dethlefsen C, Schmidt E, et al (2009) Predictive values of acute coronary syndrome discharge diagnoses differed in the danish national patient registry. J Clin Epidemiol 62: 188–194. 18722087 10.1016/j.jclinepi.2008.03.005

[25] Jensen MK, Rimm EB, Mukamal KJ, Edmondson AC, Rader DJ, et al (2009) The T111I variant in the endothelial lipase gene and risk of coronary heart disease in three independent populations. Eur Heart J 30: 1584–1589. 19411665 10.1093/eurheartj/ehp145 PMC2733737

[26] Rimm EB, Giovannucci EL, Willett WC, Colditz GA, Ascherio A, et al (1991) Prospective study of alcohol consumption and risk of coronary disease in men. Lancet 338: 464–468. 1678444 10.1016/0140-6736(91)90542-w

[27] Jensen MK, Mukamal KJ, Overvad K, Rimm EB (2008) Alcohol consumption, TaqIB polymorphism of cholesteryl ester transfer protein, high-density lipoprotein cholesterol, and risk of coronary heart disease in men and women. Eur Heart J 29: 104–112. 18063597 10.1093/eurheartj/ehm517

[28] Korn EL, Graubard BI, Midthune D (1997) Time-to-event analysis of longitudinal follow-up of a survey: Choice of the time-scale. Am J Epidemiol 145: 72–80. 8982025 10.1093/oxfordjournals.aje.a009034

[29] Kalbfleisch JD, Lawless JF (1988) Likelihood analysis of multi-state models for disease incidence and mortality. Stat Med 7: 149–160. 3353602 10.1002/sim.4780070116

[30] Rothman KJGS (1998) Modern epidemiology. Philadelphia: Lippincott Williams and Wilkins.

[31] Hosmer DW, Lemeshow S (1992) Confidence interval estimation of interaction. Epidemiology 3: 452–456. 1391139 10.1097/00001648-199209000-00012

